# Deiters’ Nucleus. Its Role in Cerebellar Ideogenesis: The Ferdinando Rossi Memorial Lecture

**DOI:** 10.1007/s12311-015-0681-9

**Published:** 2015-06-09

**Authors:** Jan Voogd

**Affiliations:** Department of Neuroscience, Erasmus Medical Center, Rotterdam, The Netherlands

**Keywords:** Deiters’ nucleus, Lateral vestibulospinal tract, Somatotopical organization, Microzones, Decerebrate rigidity

## Abstract

Otto Deiters (1834–1863) was a promising neuroscientist who, like Ferdinando Rossi, died too young. His notes and drawings were posthumously published by Max Schultze in the book “Untersuchungen über Gehirn und Rückenmark.” The book is well-known for his dissections of nerve cells, showing the presence of multiple dendrites and a single axon. Deiters also made beautiful drawings of microscopical sections through the spinal cord and the brain stem, the latter showing the lateral vestibular nucleus which received his name. This nucleus, however, should be considered as a cerebellar nucleus because it receives Purkinje cell axons from the vermal B zone in its dorsal portion. Afferents from the labyrinth occur in its ventral part. The nucleus gives rise to the lateral vestibulospinal tract. The cerebellar B module of which Deiters’ nucleus is the target nucleus was used in many innovative studies of the cerebellum on the zonal organization of the olivocerebellar projection, its somatotopical organization, its microzones, and its role in posture and movement that are the subject of this review.

Deiters’ nucleus is the target nucleus of a cerebellar module consisting of the Purkinje cells of the lateral vermal B zone. It projects to the spinal cord through the lateral vestibulospinal tract (Fig. 1). This module has been used by many scientists in the development of fundamental concepts on the function of the cerebellum, the subject of this review.

**Fig. 1:**
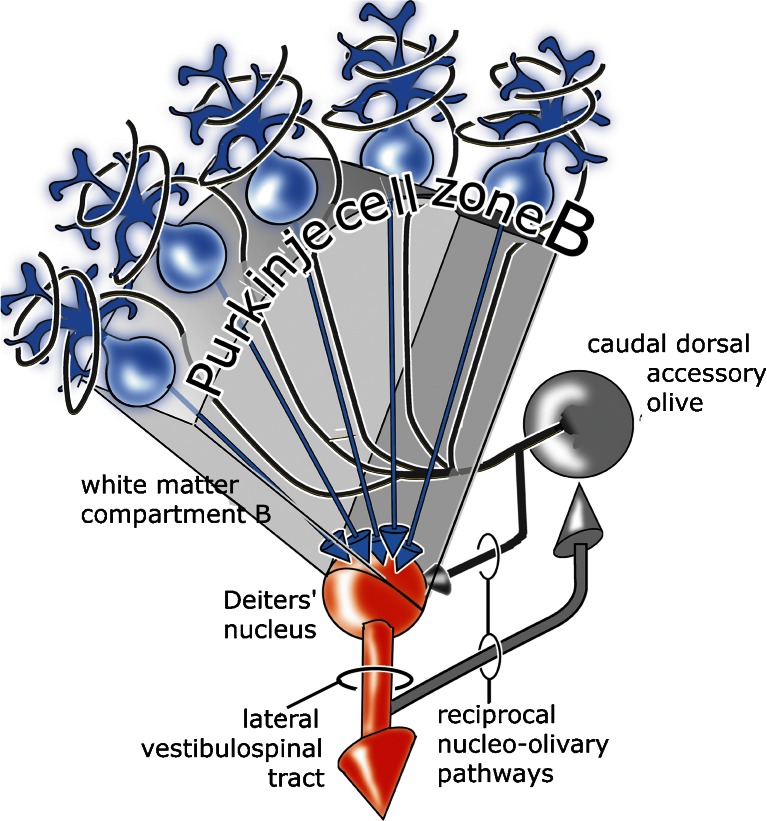
Diagram of the B module. It consists of the lateral vermal Purkinje cell zone B, Deiters’ nucleus as its target nucleus, a climbing fiber projection from the contralateral caudal dorsal accessory olive, reciprocally organized nucleo-olivary pathways, and the lateral vestibulospinal tract as its efferent pathway. The Purkinje cell axons and the olivocerebellar fibers are contained in the white matter compartment B (Fig. 7b)

Otto Deiters (1834–1863), like Ferdinando Rossi (1960–2014) to whom this paper is dedicated, died young at the age of 29 (Fig. 2). Vera Deiters and Ray Guillery [2], both descendents from Otto Deiters brother, Hermann, recently published an account of his life and work. Otto Deiters was born and spent most of his short life in Bonn. Since their father died fairly young, the brothers had to support their family. Otto Deiters practiced medicine but spend most of his time in the anatomical department of Bonn University. After his early death, professor Max Schultze and his brother collected his notes and drawings and published them in 1865 under the title “Untersuchungen über Gehirn und Rückenmark” [3]. The book is best known for Deiters’ drawings of dissected anterior horn cells with their multiple ramifying dendrites and a single axon. It also contains drawings of transverse sections through the spinal cord and the medulla of an ungulate. His Table V, an unfinished drawing of a section through the rostral medulla oblongata, is illustrated as Fig. 3. The large cells among the fiber bundles, medial to the restiform body, became known as Deiters’ nucleus.

**Fig 2:**
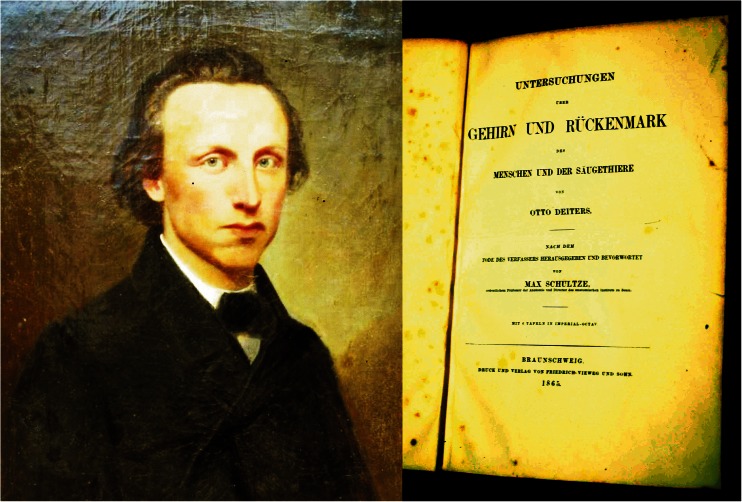
Otto Deiters (1854–1863). Portait reproduced with permission from Dr. Wolfgang and Dr. Vera Deiters, and the front page of his posthumously published book

**Fig 3:**
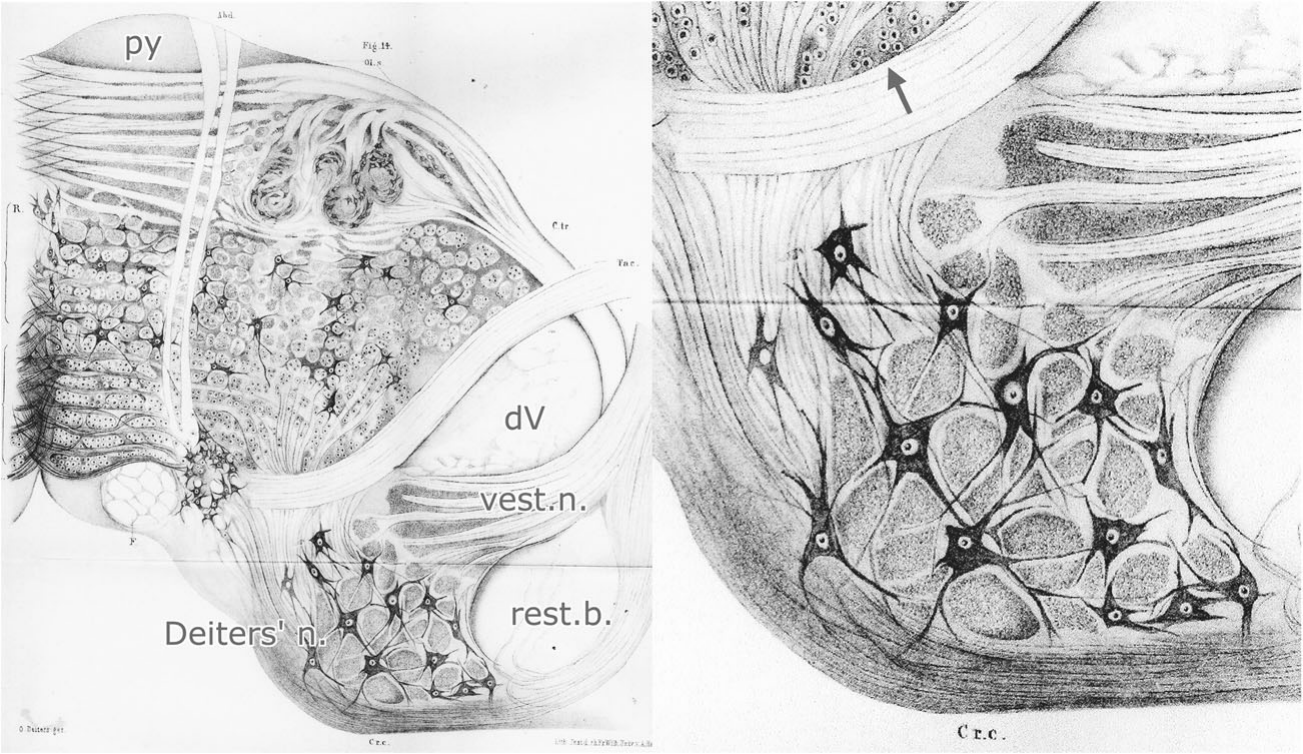
Plate V from Deiters (1865) showing the large-celled nucleus, located medial to the restiform body that bears his name. Deiters used dichromate fixation that preserves the lipids of the myelin sheath, and carmine staining. Coarse myelinated fibers are present at the *arrow. Abbreviations*: *dV* descending root of the trigeminal nerve, *py* pyramid, *rest.b*. restiform body, *vest.n*. vestibular nerve

Deiters’ description was an original one. Little was known about this region at the time. Stilling, who first illustrated serial sections through the human brainstem [4, 5], considered the bundles medial to the restiform body as a rostral continuation of the dorsal columns that enter the cerebellum more rostrally (Fig. 4a). He did not describe the large cells located among them. Clarke [6] was of the opinion that the cells located between these bundles (his “dorsal pyramid”) gave rise to the acoustic nerve, together with the ganglion of the acoustic nerve: our medial vestibular nucleus (Fig. 4b). Deiters denied a connection of his nucleus with the acoustic nerve. The origin of the vestibular and cochlear nerves from Scarpa’s ganglion and the ganglion spirale and the centripetal course of these much later nerves were established by His [7] in human embryos. Cajal [8] found Deiters’ nucleus to be heavily innervated by the vestibular nerve. In tracing studies of the vestibular nerve, it was found that its fibers avoid the large cells of Deiters’ nucleus, thus confirming Deiters’ opinion [9]. Lorente de Nó [10] stated “a region, the dorso-caudo-medial one (of the vestibular nuclei), inhabited by big cells, sometimes called the dorsal Deiters nucleus, in which no vestibular fibers penetrate: if these cells have direct connection with the vestibular nerve, it is by means of only a few collaterals. This is chiefly a cerebellar nucleus” (Fig. 5). Lorente de Nó’s use of the term “dorsal” Deiters’ nucleus is the first indication of the division of the region with large neurons in the juxtarestiform body in dorsal, cerebellar, and ventral vestibular nerve-innervated parts.

**Fig. 4:**
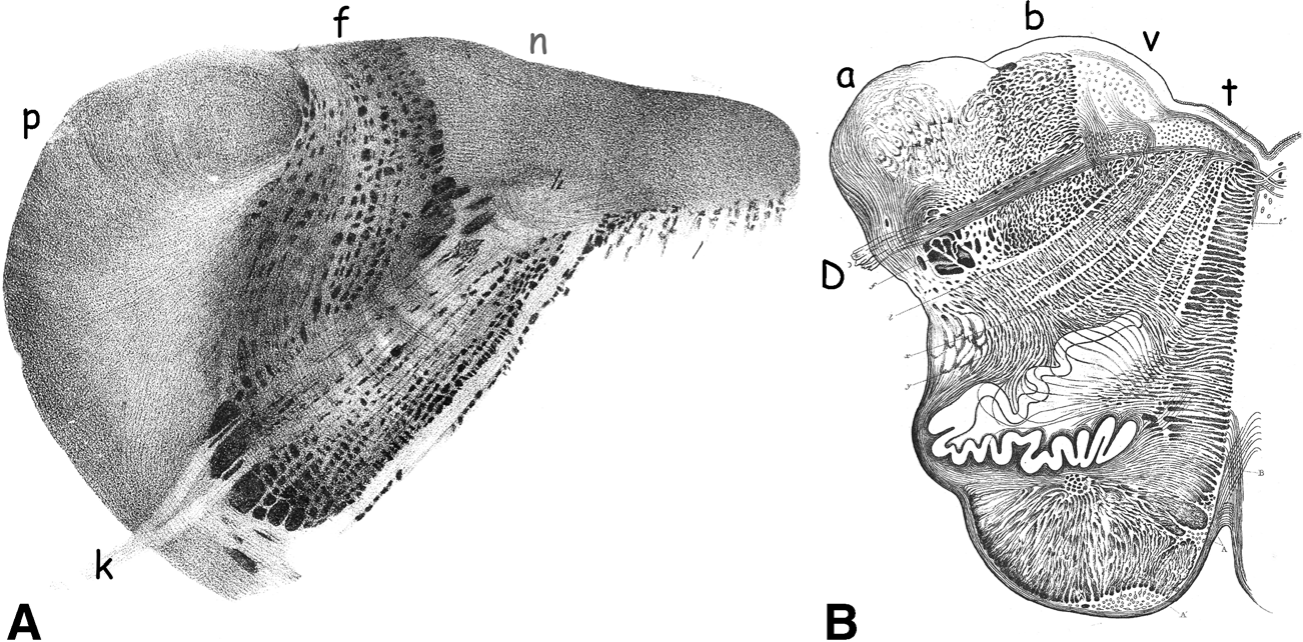
**a** Section through the dorsal medulla oblongata. Legends: *k* vagal nerve, *f* extension from the dorsal columns, *n* glossopharyngeal nucleus, *p* restiform body. Lithograph from Stilling [4]. **b** Line drawing of a section through the medulla oblongata. Legends: *a* restiform body, *b* dorsal pyramid, *D* vagal nerve, *f* nucleus of the vagal nerve, *v* ganglion of the acoustic nerve. Reproduced from Clarke [6]

**Fig. 5:**
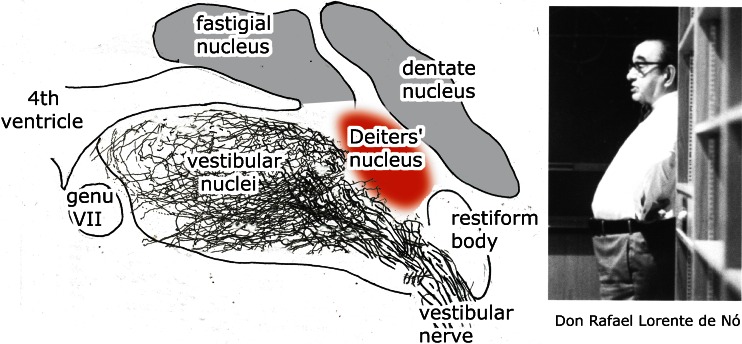
The distribution of the vestibular nerve. Stacked silver-impregnated sections from Lorente de Nó [10]. Photograph of Lorent Nó reproduced from Rodriguez and Verkhratsky [11]

The cerebellar connection of Deiters’ nucleus was first established by Klimoff in his Kazan thesis [12], published as an abstract in [13]. Klimoff traced Purkinje cell axons from the anterior vermis as “wurzelförmige Bündel” (root-like bundles) into Deiters nucleus (Fig. 6). This connection was illustrated in more detail by Hohman [1]. He distinguished two discrete bundles of Purkinje cell axons in the white matter of the anterior vermis, the “medial anterior vermis-fastigius bundle” and the “lateral anterior vermis-Deiters bundle” (Fig. 7a). This observation was extended and confirmed by Voogd [14, 15] who delineated compartments in the cerebellar white matter in cat and ferret that contain the axons from longitudinal Purkinje cell zones on their way to their target nuclei. The medial A zone provides the projection to the fastigial nucleus, contained in the A compartment. Axons from the lateral B zone populate the B compartment that corresponds to Hohman’s lateral anterior vermis-Deiters bundle (Fig. 7b). These observations on the corticonuclear projection and the localization of the B zone were extended to primates and marsupials by Haines (for a review see [16]).

**Fig. 6:**
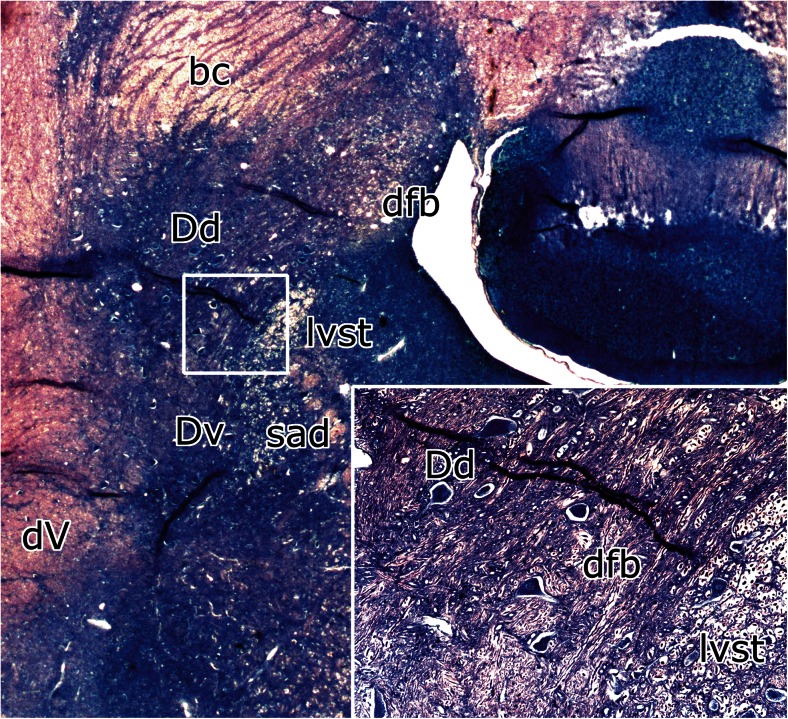
Häggqvist-stained section through Deiters’ nucleus of the cat. Purkinje cell axons pass through the brachium conjunctivum (Klimoff’s “Wurzelförmige Bündel”) to enter dorsal Deiters’ nucleus. Dichromate fixation makes it possible to distinguish the coarse myelinated fibers of the direct fastigiobulbar tract and the lateral vestibulospinal tract (inset). *Abbreviations*: *bc* brachium conjunctivum, *Dd* dorsal Deiters’ nucleus, *dfb* direct fastigiobulbar tract, *dV* spinal root of the trigeminal nerve, *Dv* ventral Deiters’ nucleus, *lvst* lateral vestibulospinal tract, *sad* dorsal acoustic striae

**Fig. 7:**
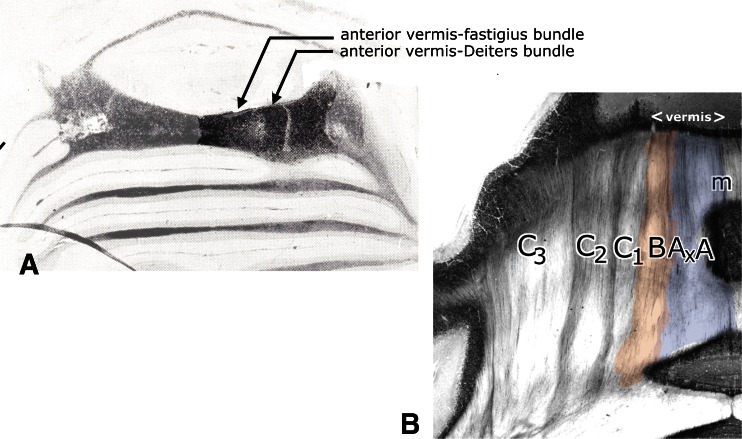
**a** Hohman [1] distinguished three discrete bundles of degenerated Purkinje cell axons in the white matter of the anterior vermis after lesions of the cortex of this area (Marchi method). The medial anterior vermis-fastigius bundle and the lateral anterior vermis-Deiters bundle are located in the vermis and terminate in these nuclei. **b** White matter compartments in the anterior lobe of the monkey cerebellum. The borders of the compartments A-D stain for acetylcholinesterase. The A (with Ax) and B compartments contain the Purkinje axons of Hohman’s anterior vermis-fastigius- and Deiters bundles, respectively

Experimental studies showed that the projection of the anterior vermis to Deiters’ nucleus is more complicated: apart from the projection of the B zone, a contingent of Purkinje cell axons of the A zone proceeds, beyond the fastigial nucleus, to terminate in Deiters’ nucleus [17–19]. Retrograde labeling from Deiters’ nucleus resulted in the presence of two strips of labeled Purkinje cells in the anterior vermis. The wide, lateral strip corresponds to the B zone; the narrow medial strip occupies a position in the A zone. In the dorsal anterior lobe, they are separated by the zebrin-negative Purkinje cells of the Ax and X zones (Fig. 8). Labeling of the medial strip was produced by a more ventral injection involving the ventral part of Deiters nucleus and the medial vestibular nucleus. In the rat, this strip occupies a position in between the zebrin-positive bands 2 and a [20]. Apart from the vestibular nuclei, its Purkinje cell axons, presumably, also terminate along the lateral border of the fastigial nucleus.

**Fig. 8:**
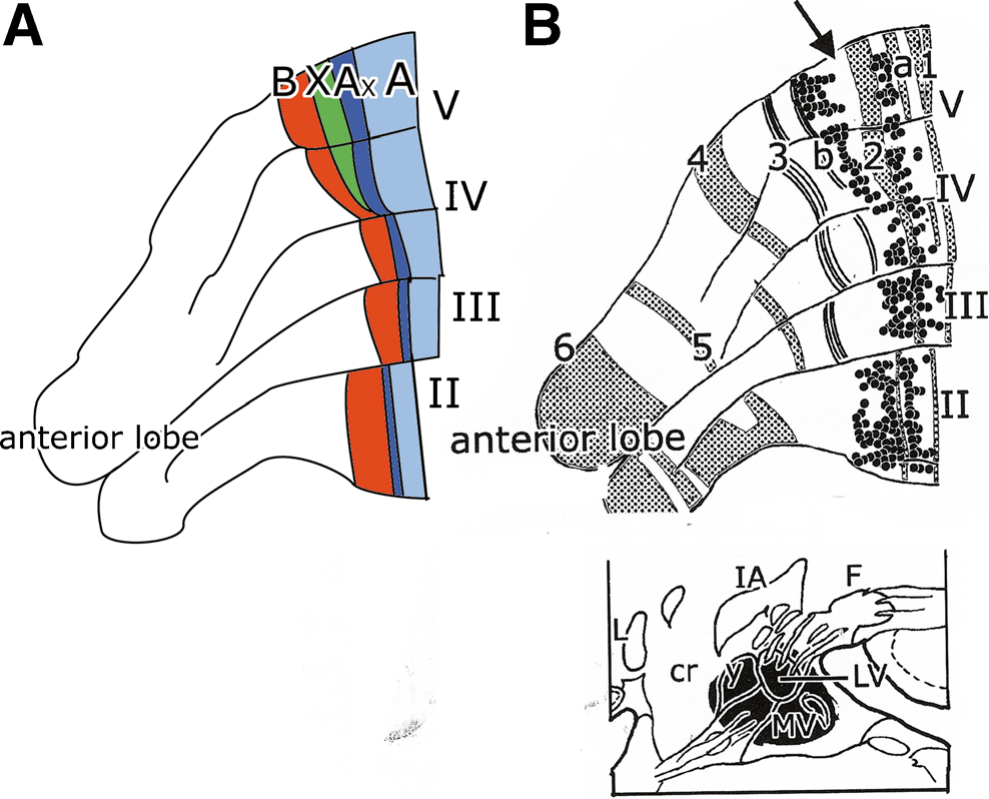
**a** Purkinje cell zones in the anterior vermis of the rat cerebellum. **b** A reconstruction of the anterior lobe of the rat showing the location of retrogradely labeled Purkinje cells from an injection of Deiters’ nucleus. Zebrin-positive bands are *shaded*. The Purkinje cells of the B zone, and a narrow strip of Purkinje cells in the A zone, located between zebrin-positive *band 2* and *a* are labeled. The Ax zone corresponds to the zebrin *band 2* in *B*. The X zone is located in the zebrin-negative region, immediately lateral to zebrin-positive *band 2* (*arrow*). *Abbreviations*: *cr* restiform body, *F* fastigial nucleus, *IA* anterior interposed nucleus, *LV* Deiters’ nucleus, *MV* medial vestibular nucleus

The origin of the lateral vestibulospinal tract (LVST) from Deiters’ nucleus was established by von Monakow [21] from chromatolysis of its neurons from a hemitransection of the cervical cord in a neonatal rabbit. Its coarse myelinated fibers descend in the reticular formation (Fig. 6) to a ventral position in the ventrolateral funiculus of the spinal cord, where it descends to coccygeal levels. Two other vestibulospinal tracts descend in the medial longitudinal fascicle, the ipsilateral medial vestibulospinal tract (MVST) and its crossed component, also known as the crossed vestibulospinal tract (CVST) [22]. Like the LVST, the CVST contains coarse fibers. Smaller fibers predominate in the MVST. According to Akaike et al. [23], inhibitory components of the vestibulospinal tracts conduct at a slower velocity. The MVST, indeed, is an inhibitory, presumably glycinergic, system. The CVST contains an inhibitory component from the posterior semicircular canal [24]. Both tracts descend in the ventral funiculus to thoracic levels.

A clear distinction between the origin of the LVST and the MVST and the CVST only was made fairly recently in the retrograde labeling experiments of Shinoda et al. [25]. They sectioned the medial longitudinal fascicle or the LVST before applying the retrograde tracer to the spinal cord. The LVST is an uncrossed descending tract that takes its origin from the lateral vestibular nucleus of Deiters and the adjoining descending vestibular nucleus. The MVST and the CVST arise from the bilateral areas where the medial, lateral, and descending vestibular nuclei meet (Fig. 9). It should be emphasized that Deiters’ nucleus, as delineated by Shinoda, includes both a dorsal, B-zone-innervated and a ventral region where fibers of the vestibular have been found to terminate.

**Fig. 9:**
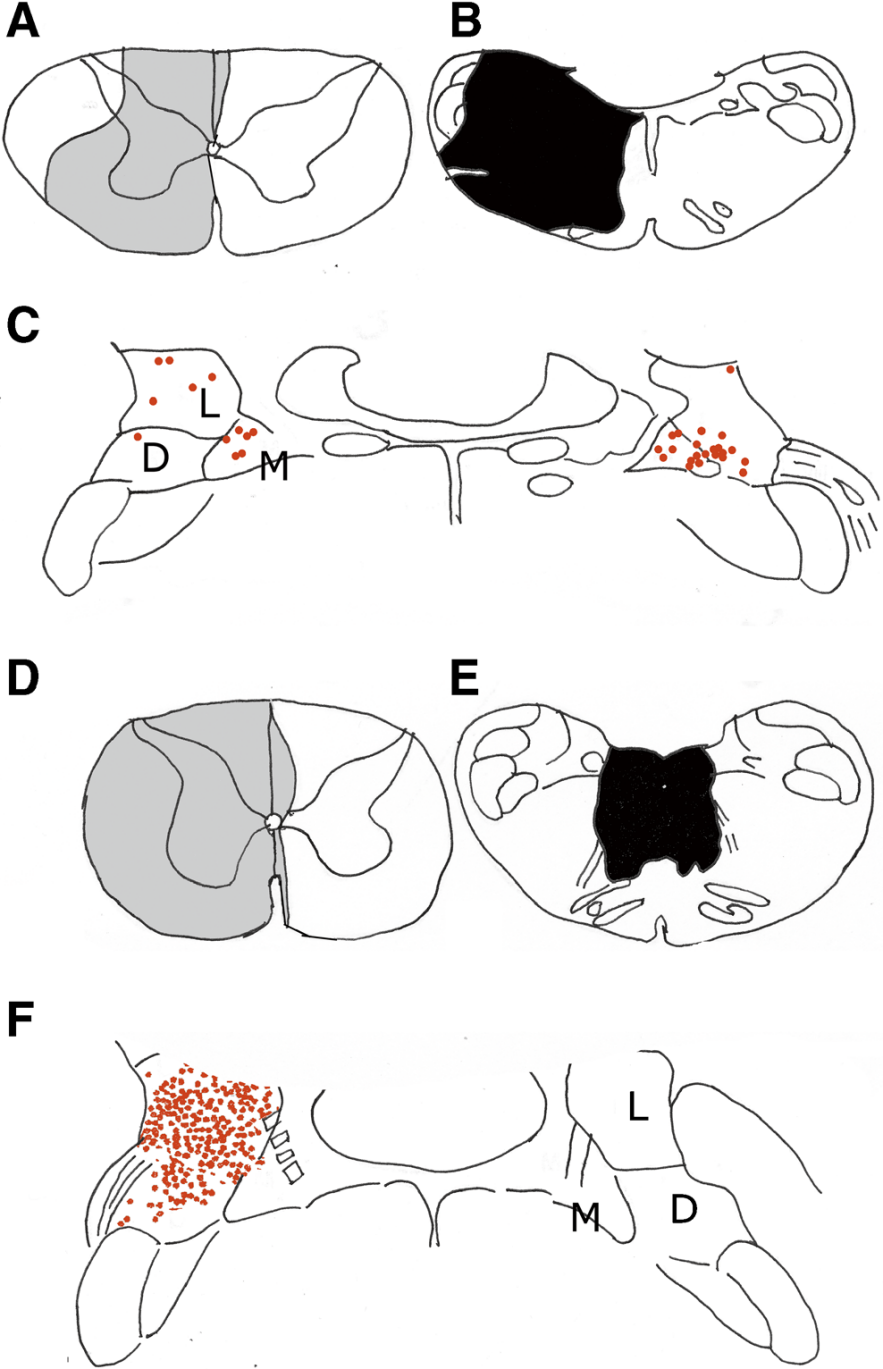
The origin of the MVST and the CVST was determined in the cat by an injection of a retrograde tracer at C1 (**a**) and a lesion of the left medulla oblongata, including the LVST (**b**). The origin of the LVST (**f**) was found, using the same method, but with a lesion of the medial longitudinal fascicle Redrawn from Shinoda et al. [25]. *Abbreviations*: *D* descending vestibular nucleus, *L* lateral vestibular (Deiters’) nucleus, *M* medial vestibular nucleus

Studies of the projection of Deiters’ nucleus along the LVST to the spinal cord have been complicated by the inclusion by most authors of both parts of the nucleus. Tracing studies that were limited to the dorsal, B-zone-innervated part never has been published. An influential example was Brodal and Pompeiano’s [26] description of the vestibular nuclei of the cat (Fig. 10). Their lateral vestibular nucleus includes both the larger and smaller neurons in a more ventral and rostral region, that receives the vestibular nerve and the dorsal and caudal group of large neurons located among the Purkinje cell axons of the B zone. A somatotopical organization of Deiters’ nucleus was described by the same authors [27]. Purkinje axons of the B zone terminate predominantly in the hindlimb portion of the nucleus, whereas its forelimb region is dominated by vestibular afferents.

**Fig. 10:**
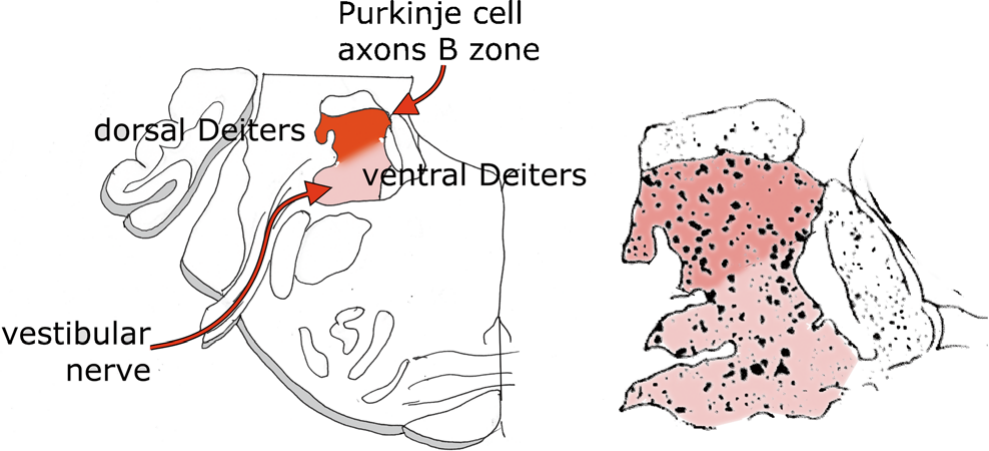
The lateral vestibular (Deiters’) nucleus. Modified from Brodal and Pompeiano [26]. They included both the dorsal, B zone-innervated and the ventral, vestibular-innervated parts in their lateral vestibular nucleus

Although dorsal and ventral Deiters nucleus can be distinguished by their Purkinje cell and vestibular input, the border or the amount of overlap between the projections of the two afferent systems is not clear. This problem was analyzed in more detail in electrophysiological studies to be discussed below. Other cerebellar system that impinge on this region stem from the A zone and from the fastigial nucleus: the crossed uncinate tract that decussates in the cerebellar commissure, and the direct fastigiobulbar tract that passes along the lateral border of the fourth ventricle to enter the vestibular nuclear complex. The uncinate tract terminates in ventral Deiters’ nucleus, the descending vestibular nucleus, and the reticular formation. The termination of the direct fastigiobulbar tract is very similar but less extensive [28, 29] (Fig. 11). Mossy fiber collaterals of the lateral reticular nucleus, and possibly from the spinocerebellar tracts, terminate in dorsal Deiters’ nucleus [30].

**Fig. 11:**
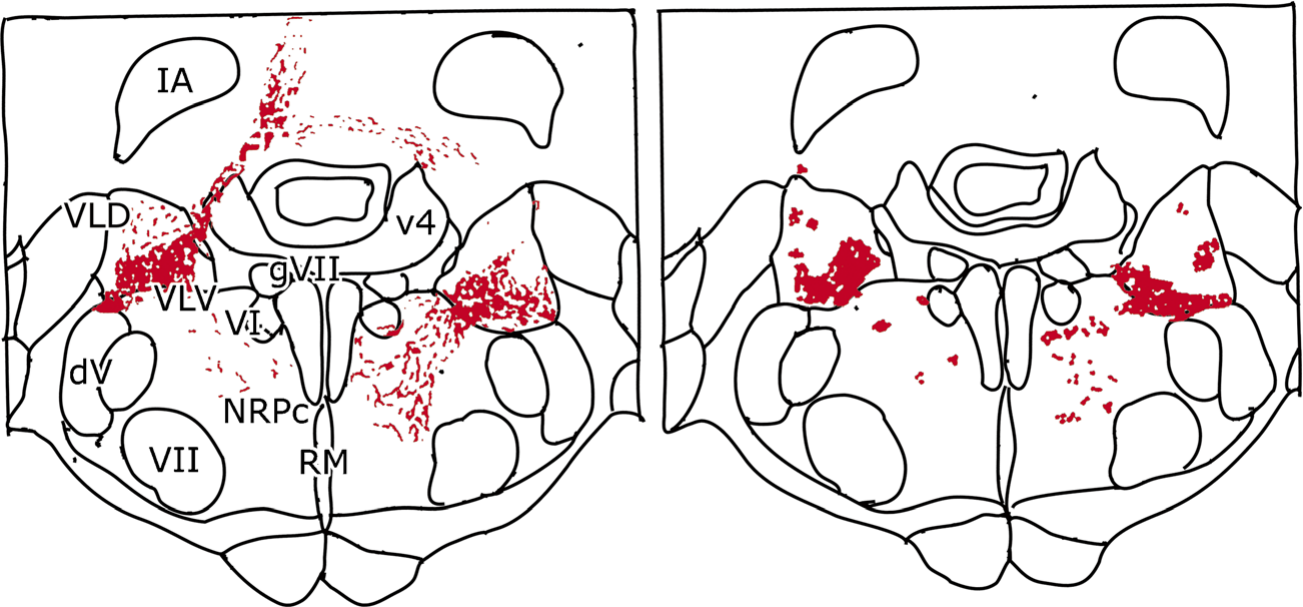
Diagram of the termination of the direct fastigiobulbar tract (left side of both panels) and the crossed uncinate tract (right side) in the cat. *Left panel* represents the axons traced with Phaseolus leucoagglutinin injected in the fastigial nucleus. In the *right panel*, the terminal boutons are plotted. Redrawn from Homma et al. [29]. *Abbreviations*: *IA* anterior interposed nucleus, *VLD* dorsal Deiters’ nucleus, *DLV*, ventral Deiters’ nucleus, *V4* fourth ventricle, *VI* trochlear nucleus, *NRPc* nucleus reticularis pontis caudalis, *RM* nucleus raphes magnus, *dV* descending root of the trigeminal nerve, *gVII* genu of the facial nerve, *VII* facial nucleus

The LVST terminates in the ipsilateral ventromedial ventral horn (laminae VIII and adjacent VII). Collaterals were found in medial motoneuronal groups [31–33] (Fig. 12). At cervical levels, collaterals also invade the lateral parts of the ventral horn. At thoracic levels, they spread over the entire ventral horn. At lumbar levels, they are restricted to the laminae VIII and adjacent VII. The large number of papers on the terminations of the tract is referenced in the papers of Liang et al. [34], Nyberg-Hansen and Mascitti [35], Petras [36], and Zemlan et al. [37]. However, in none of these papers, the projections of dorsal and ventral Deiters’ nucleus were distinguished.

**Fig. 12:**
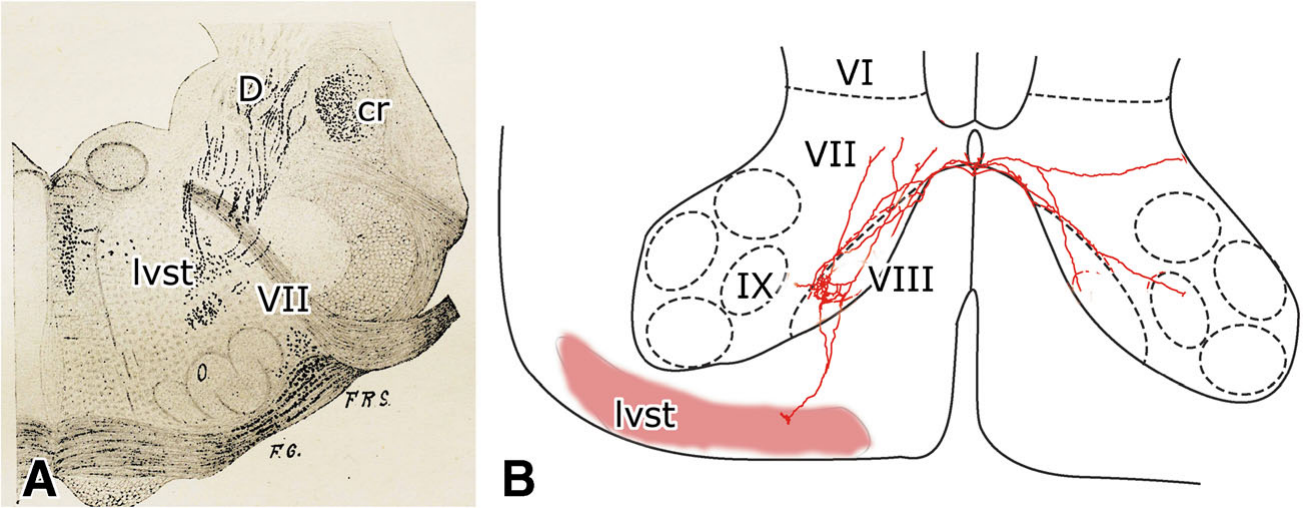
**a** The lateral vestibulospinal tract, passing along the facial nerve to become located in the center of the reticular formation. Reproduced from van Gehuchten [31]. **b** Collaterals of the lateral vestibulospinal tract terminating in the cervical cord. Modified from Shinoda et al. [25]. *Abbreviations*: *cr* restiform body, *D* Deiters’ nucleus, *lvst* lateral vestibulospinal tract, *VII* facial nerve

One of the components of a cerebellar module is the climbing fiber projection from a subdivision of the inferior olive to its Purkinje cells, with a collateral projection to their target nucleus. The longitudinal zonal termination of climbing fibers was discovered by Oscarsson and Uddenberg [38]. They stimulated hind- and forelimb nerves in cats with a transection of the cord except for the ventral funiculus that contains the ventral funiculus olivocerebellar climbing fiber path (vfSOCP). They recorded climbing fiber potentials from the lateral anterior vermis, located in a longitudinal strip, later identified as the B zone (Fig. 13). Responses from stimulation of either the left or right hindlimb nerves were located lateral to those evoked from forelimb nerves. The origin of the climbing fiber projection to the B zone later was established in the caudal dorsal accessory olive [15, 39].

**Fig. 13:**
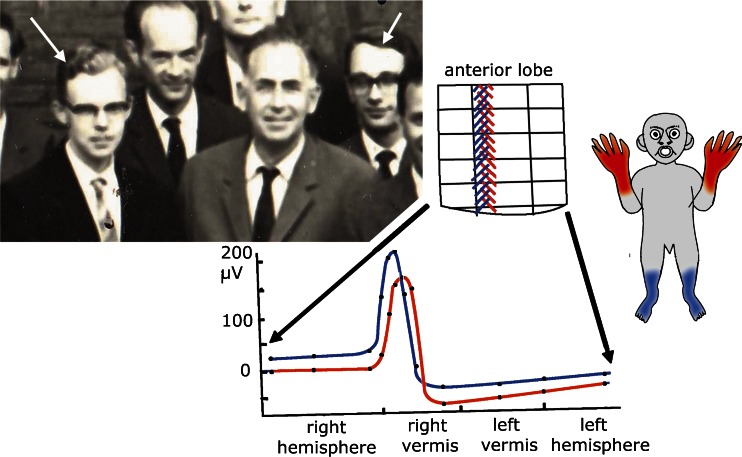
Diagram of the medio-lateral somatotopical organization of climbing fiber evoked potentials in what later was called the B zone of the anterior lobe of the cat. *Red* stimulation of right dorsal radial nerve. *Blue* stimulation of right hamstring nerve. *Curves* show amplitude of surface potentials recorded along a single folium. Redrawn from Oscarsson and Uddenberg [38]. Inset: Olav Oscarsson (*left*) and the author (*right*) at a meeting at the time of publication of this paper

A similar medio-lateral somatotopical organization of mossy fiber projections to the B zone can be reconstructed from the work of Ji and Hawkes [40]. They plotted mossy fiber terminals from the spinocerebellar tracts, labeled from the thoracic cord, i.e., systems that carry information from the hindlimb, and the forelimb cuneocerebellar tract, relative to the position of zebrin-positive and zebrin-negative bands. Because the B zone in lobule III of the anterior lobe of the rat is known to be situated lateral to zebrin-positive band 2 (Fig. 8), hind- and forelimb mossy fibers can be seen to terminate in lateral and medial parts of the B zone, respectively (Fig. 14). The medio-lateral somatotopical organization of the vermal B zone differs from the mainly rostro-caudal somatotopical organization of the hemisphere, as established by Snider and Stowell [41], Adrian [42], and Snider and Eldred [43] (Fig. 15).

**Fig. 14:**
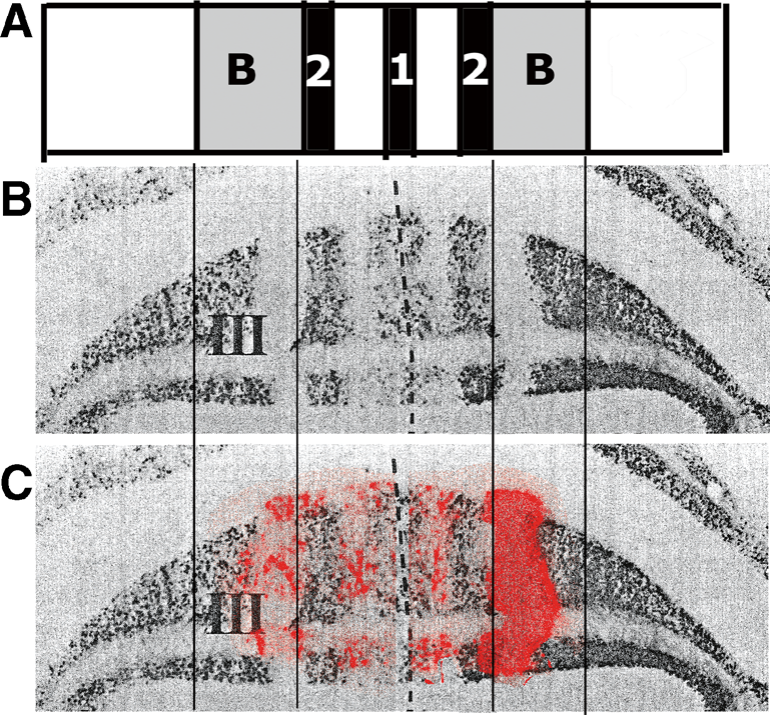
Medio-lateral somatopical organization of mossy fibers in the B zone of rat cerebellum. **a** Diagram showing location of B zone relative to zebrin-positive band P2+ (compare Fig. 8). **b** Spinocerebellar mossy fibers traced with horseradish peroxidase from an injection of the thoracic cord (hindlimb). **c** Cuneocerebellar terminals traced from an injection of the cuneate nucleus (forelimb) in *red*, superimposed on **b**. Data reproduced from Ji and Hawkes [40]

**Fig. 15:**
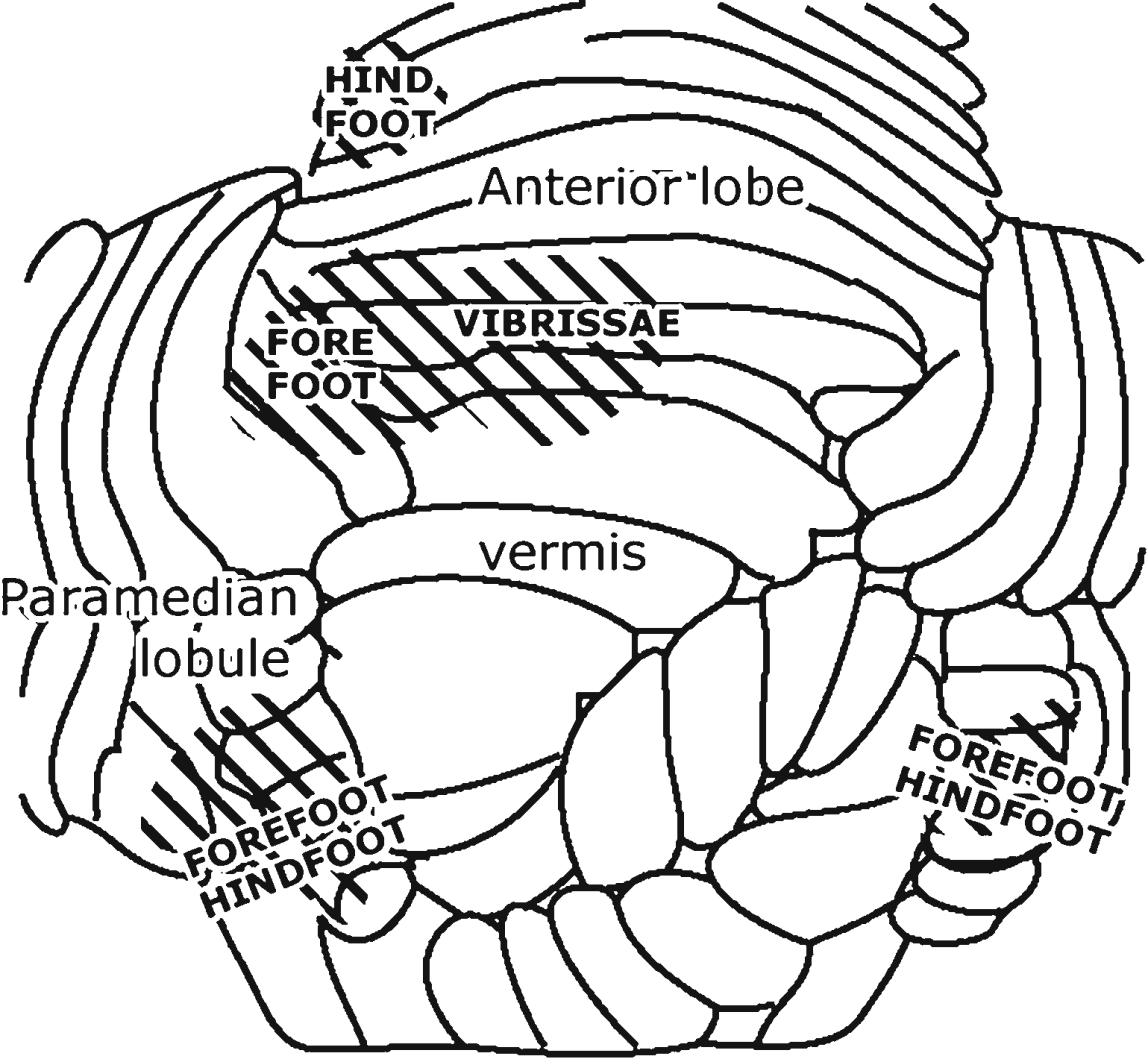
Tactile projection areas in the cerebellar cortex of fore- and hindfoot of the cat. Mirrored location in the hemisphere of the anterior lobe and the paramedian lobule. Redrawn from Snider and Stowell [41]

The medio-lateral somatotopical organization of the B zone was confirmed and extended by Andersson and Oscarsson [44, 45]. They recorded climbing fiber potentials from Purkinje cells of the B zone and found a regular arrangement of long, narrow “microzones” containing Purkinje cells that receive their climbing fiber input from the same receptive fields. Responses on stimulation of hindlimb nerves are located in the lateralmost microzone, purely forelimb in a medial microzone and different combinations in the intermediate three microzones (Fig. 16). Each of these microzones projects to a particular population of neurons in Deiters’ nucleus. These neurons are intermingled in the nucleus, with the hindlimb population predominating in its caudal part. The somatopical organization of the nucleus, apparently, is not as precise as maintained by Pompeiano and Brodal [27]. A minor projection of the A zone to Deiters’ nucleus was noticed in their experiments. The authors considered the microzone with their target neurons as the basic computational unit of the cerebellum.

**Fig. 16:**
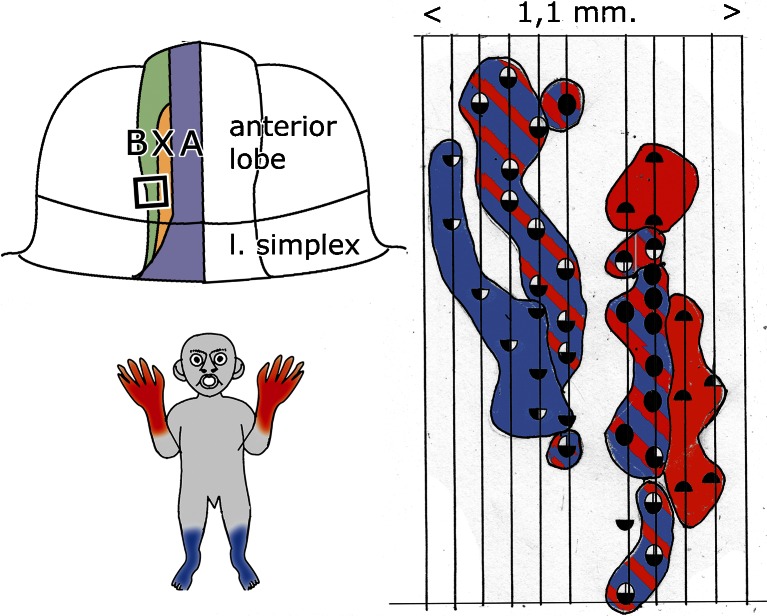
Distribution and climbing fiber response patterns of Purkinje cells in the B zone of the anterior lobe of the cat. Purkinje cells with identical climbing fiber responses are arranged in microzones: Medialmost microzone receives climbing fiber input from ipsilateral sciatic nerve, the lateralmost microzone from the ipsi- and contralateral ulnar nerves. Intermediate microzones are innervated by both fore- and hindlimb nerves. Redrawn from Andersson and Oscarsson [45]

Some physiologists distinguished between the dorsal, cerebellar and ventral, vestibular innervated divisions of Deiters’ nucleus. The most detailed account stems from Akaike’s papers on rabbit [23] and cat [46]. Figure 17 shows the distribution in the cat of “second-order” neurons of the cat LVST and MVST that are activated by stimulation of the vestibular nerve, and the “non-second-order” neurons, presumably activated by Purkinje cell axons of the B zone. In the sagittal sections of Fig. 17, the LVST non-second-order neurons predominate in the dorsal Deiters’ nucleus. Second-order neurons are located in ventral Deiters and, together with MVST neurons, in the descending vestibular nucleus. The LVST second-order neurons project in equal numbers to all levels of the spinal cord. Of the LVST non-second-order neurons, about half projects to the lumbar cord. An even higher percentage of 78 % was found for these LVST non-second-order neurons by Wilson and Yoshida [47]. It can be concluded that the influence of the B-zone-innervated dorsal Deiters’ nucleus is preferentially exerted on the hindlimb, whereas ventral Deiters’ nucleus, with its mixed vestibular and cerebellar input and its output through the LVST, the MVST, and the CVST, is mainly focussed on the forelimb. But what are the effects of the LVST in the lumbar cord?

**Fig. 17:**
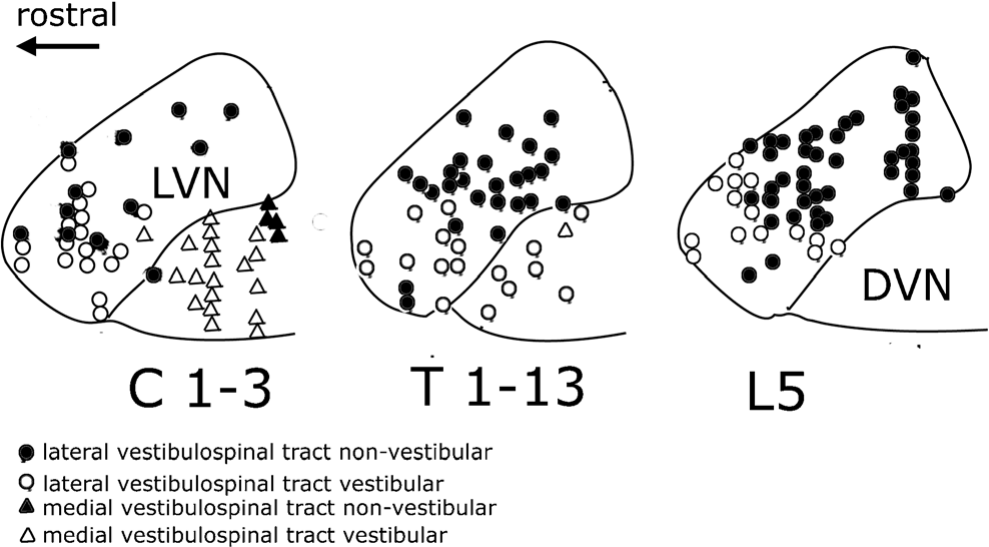
Neurons activated from stimulation of the vestibular nerve in the cat (second-order neurons: *open symbols*) and neurons not activated by the vestibular nerve, presumably innervated by Purkinje cell axons of the B zone (non-second-order neurons: *filled symbols*) that give rise to the lateral vestibulospinal tract (*circles*) and the medial vestibulospinal tracts (*triangles*) plotted on a sagittal section through Deiters’ lateral vestibular nucleus (LVN) and the descending vestibular nucleus (DVN). Reproduced from Akaike (1983)

Purkinje cells of the B zone and Deiters’ nucleus are involved in the control of both extensor and flexor muscles of the lower limb as shown by the transneuronal retrograde labeling of these structures from injections of rabies virus in these muscles in the rat [48]. Stimulation of Deiters’ nucleus in decerebrate cats causes mono- and disynaptic excitatory postsynaptic potentials (EPSP’s) in ipsilateral alpha and gamma extensor motoneurons [47] and disynaptic inhibitory PSP’s in flexor motoneurons, transmitted by Ia inhibitory interneurons (Fig. 18). Monosynaptic connections are mainly with ankle and knee extensors [49–51]. Similar synaptic connections are present on the contralateral side. Disynaptic EPSP’s in contralateral extensor motoneurons are mediated by commissural interneurons, located in lamina VIII of the lumbar cord. Trisynaptic inhibition of contralateral flexor motoneurons is mediated by commissural neurons and Ia inhibitory interneurons [52, 53].

**Fig. 18:**
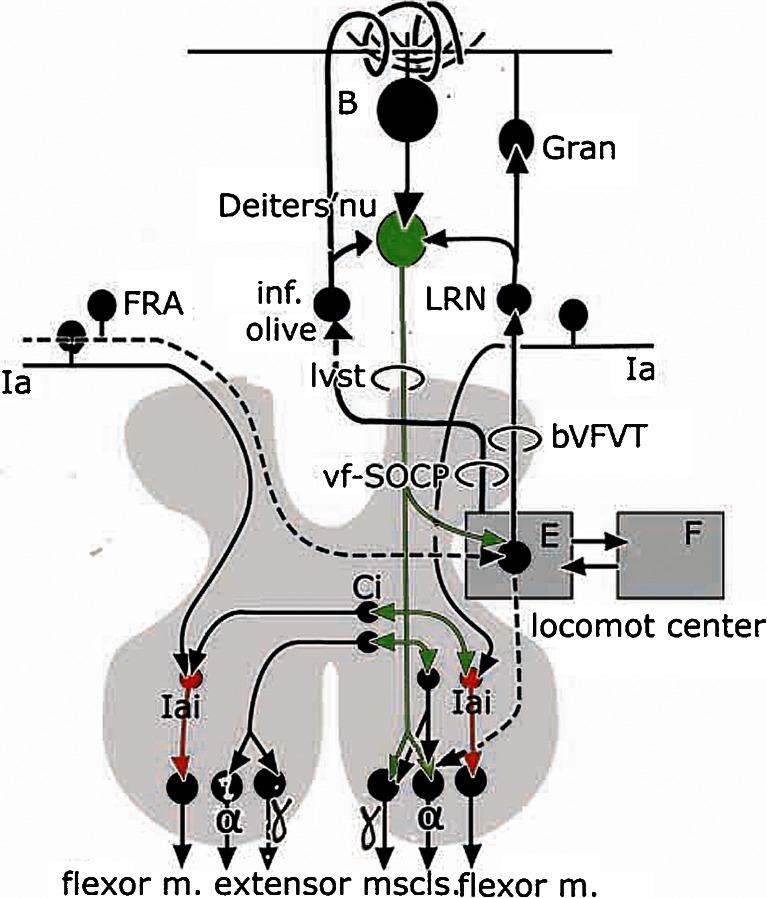
Diagram of the synaptic connections of the lateral vestibulospinal tract. Polysynaptic pathways are shown as *broken lines. Abbreviations*: *B* Purkinje cells of the B zone, *bVFRT* bilateral flexor reflex tract, *Ci* commissural interneuron, *E* extensor half center, *F* flexor half center, *FRA* flexor reflex afferents, *Gran* granule cell, *Ia* Ia afferents, *Iai* Ia inhibitory interneuron, *LRN* lateral reticular nucleus, *lvst* lateral vestibulospinal tract, *vfSOCP* ventral funiculus spino-olivary climbing fiber path

Deiters’ nucleus also has important effects on locomotion. Stimulation of the nucleus increases gastrocnemius muscle activity when applied during late swing phase and standing [54]. Spike frequency in forelimb cells in Deiters’ nucleus in decerebrate cats was modulated with peaks in the late stance and the swing phase of the ipsilateral forelimb. Obstruction of the paw during stance shortened the ongoing stance and swing phase and spike frequency in the contralateral Deiters’ nucleus increased [55]. According to these authors, spike modulation in Deiters’ nucleus is controlled by Purkinje cells of the B zone. Perturbations during walking, indeed, evoke complex spikes in the Purkinje cells of the B zone [56]. Stimulation of Deiters’ nucleus was found to reset locomotion rhythm [57]. LVST and contralateral flexor reflex afferents (FRA) from wide receptive fields were found to converge, via a polysynaptic pathway, upon interneurons in the extensor half center of the central pattern generator by Leblond et al. [58]. Facilitation of LVST-induced EPSP’s in extensor motoneurons by stimulation of contralateral FRA earlier was shown by Ten Bruggencate et al. [51] and Grillner et al. [59].

Stimulation of LVST activates reciprocal ascending FRA pathways such as the bilateral ventral flexor reflex tract (bVFRT) and the a and b subsets of the ventral funiculus spino-olivocerebellar climbing fiber path (a and b vf-SOCP) (Fig. 18). The bVFRT terminates in the lateral reticular nucleus, a nucleus that provides Deiters’ nucleus with a collateral innervation and the vermis with mossy fiber afferents [30, 60]. The a and b vf-SOCP provide the A and B zones with their climbing fibers via the caudal medial and dorsal accessory olives and, similarly, provide the fastigial and Deiters’ nuclei with a collateral innervation [39, 61]. In Fig. 18, these systems are shown to originate from neurons in the extensor half of the locomotion center where LVST and contralateral FRA converge, but as yet their precise origin is not known. These FRA pathways may carry information related to interneuronal activity in segmental motor centers to the cerebellum as has ben suggested by Lundberg [62] and Oscarsson [63].

One of the longest stories in cerebellar ideogenesis is the cerebellar inhibition of decerebrate rigidity. Decerebrate rigidity was first observed by Sherrington [64] in monkeys after removal of the cerebral hemispheres (Fig. 19). “If a finger or one of the monkeys hands be stimulated … there results an extensive reflex reaction involving movement of the entire upper limb. The wrist is extended, the elbow flexed, the shoulder protracted. … The most striking feature of the reflex is, however, that when the actual movement has been accomplished, the contraction of the muscles employed in it does not cease or become superseded by the action of another group, but is continued even for 10 or 20 min at a time … Analogous results are obtainable on the hindlimb.” Sherrington called it “the cataleptoid reflex.” The same year, Löwenthal and Horsley [65] studied decerebrate rigidity in cats and dogs, the animals used in most subsequent studies (Fig. 20). They made “the observation that when both cerebral hemispheres were removed, and as a result, acerebral tonus (i.e., decerebrate rigidity JV) of the limbs was obtained, excitation (faradic) of the upper surface of the cerebellum caused immediate relaxation of such tonus.” Thiele [66] found in decerebrate cats that “unilateral separation of Deiters’ nucleus from the medulla or the division of the (lateral vestibulospinal JV) tract as it passes down through the medulla produced relaxation of the rigidity on the homolateral side”: the first mention of the role of Deiters’ nucleus in the production of decerebrate rigidity. Inhibition of decerebrate rigidity usually was studied in postcollicular decerebrated cats that produces permanent extension of all four extremities, with some opisthotonus. Cerebellar regions that were responsible for the relaxation of decerebrate rigidity when stimulated were determined by Bremer [67], professor of physiology at the Université Libre de Bruxelles. He localized the effective zones in the anterior vermis and, less effectively, in the pyramis of the caudal vermis (Fig. 21).

**Fig. 19:**
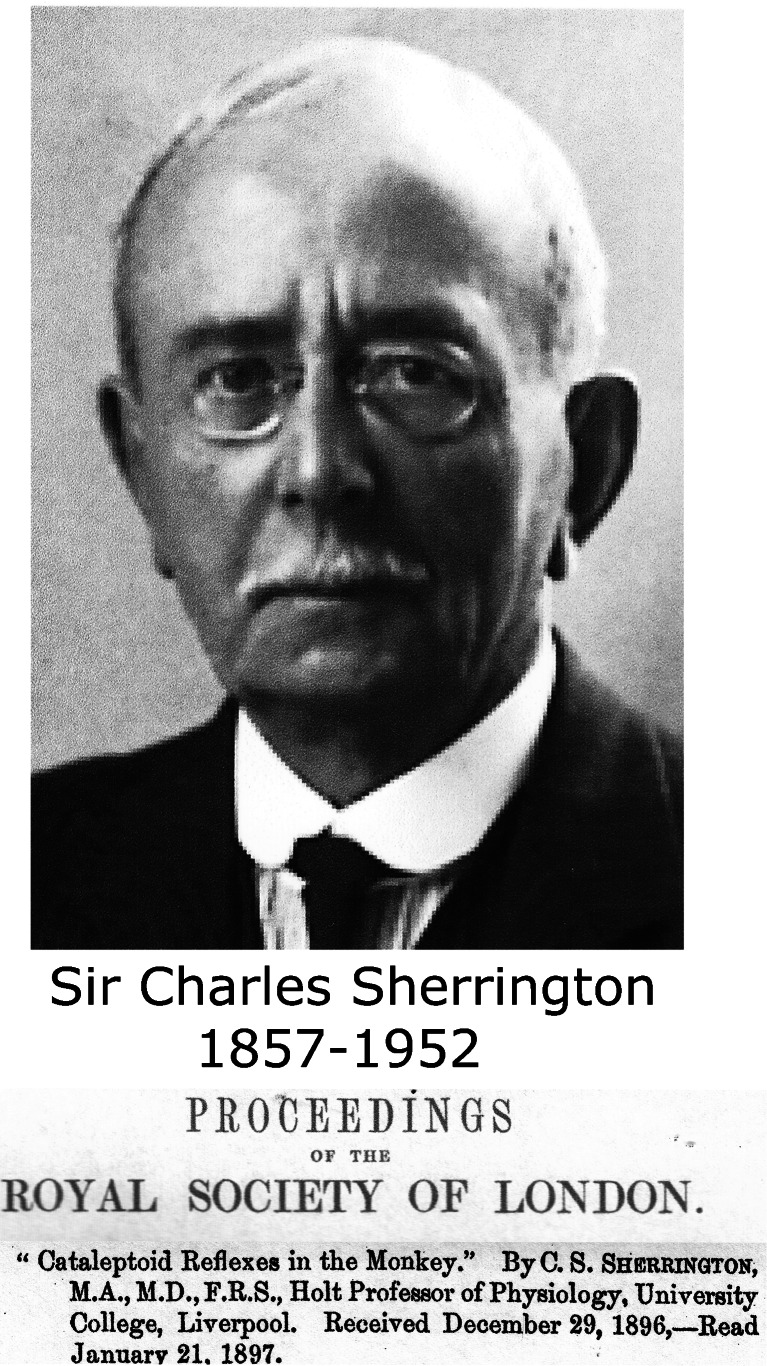
Sir Charles Sherrington: the first (897) description of decerebrate rigidity

**Fig. 20:**
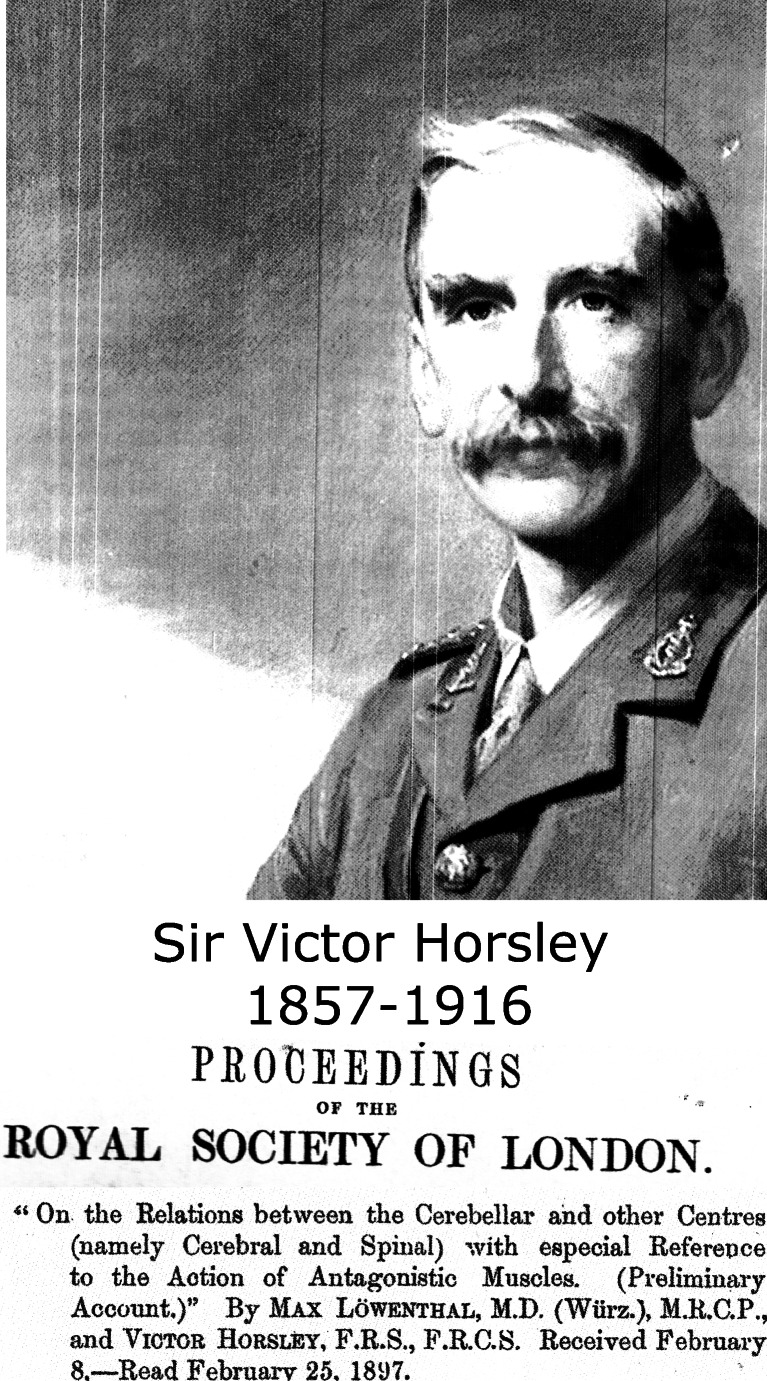
Sir Victor Horsley published the first observation of cerebellar inhibition of decerebrate rigidity, together with Max Löwental (1897)

**Fig. 21:**
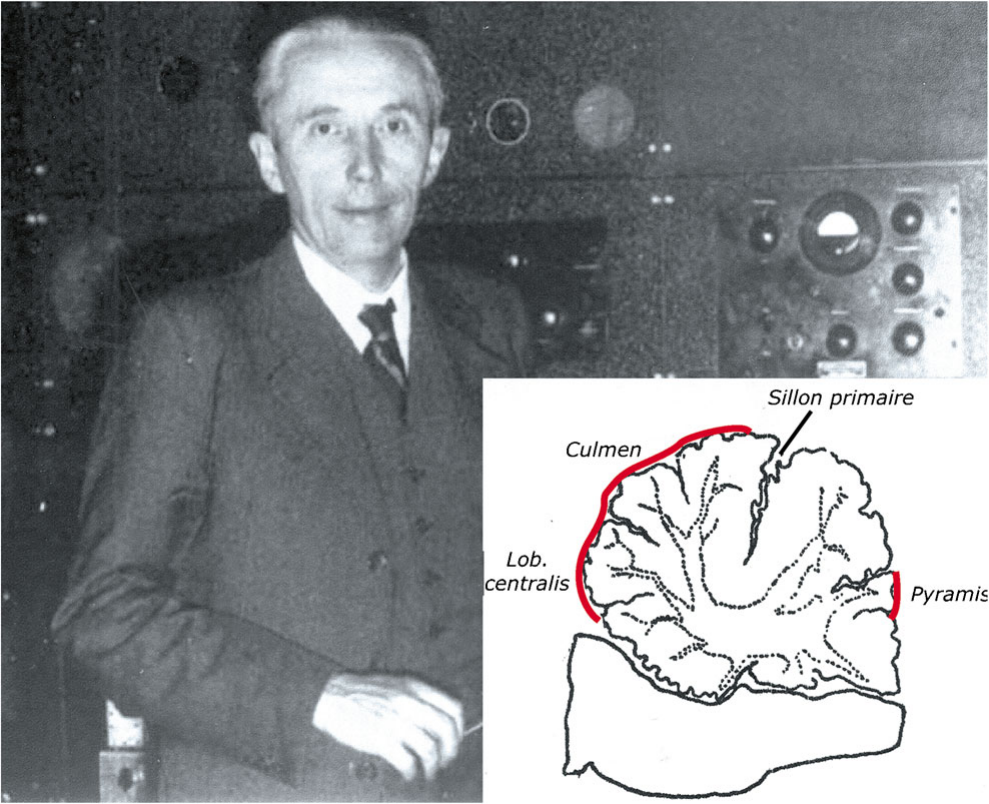
Frédérique Bremer (1892–1982). Professor of physiology at the université Libre de Bruxells. Inset shows the effective regions for inhibition of decerebrate rigidity in the cat. Redrawn from Bremer [67]. Photograph reproduced from Kerkhofs and Lavie [68]

Bremer’s studies were continued by Moruzzi, whose interest in cerebellar physiology was raised during a postdoc with Bremer. Moruzzi authored an extensive review of pre-PubMed cerebellar physiology, an indispensable source of information for those interested in the roots of cerebellar neurobiology ([69], Fig. 22). In this book, he summarized the following experiments he did in collaboration with Pompeiano. He repeated the experiments on inhibition of decerebrate rigidity in decerebrate cats by stimulation of the anterior vermis. As to be expected, removal of the anterior vermis caused an increase of the extensor tone. A lesion of the rostral fastigial nucleus caused ipsilateral relaxation of extensor tone: Moruzzi’s “ipsilateral fastigial atonia” (Fig. 23). A lesion of the caudal fastigial nucleus produced contralatral relaxation of extensor tone: the “crossed fastigial atonia.” Bilateral ablation of the caudal fastigial nucleus restored the extensor tone in all limbs. The results from his experiments on the anterior vermis could be explained by the stimulation or ablation of the B-zone-Deitersian system. His observations on lesions of the rostral fastigial nucleus and the recurrence of rigidity after bilateal lesions of the caudal fastigial nucleus are much more difficult to explain.

**Fig. 22:**
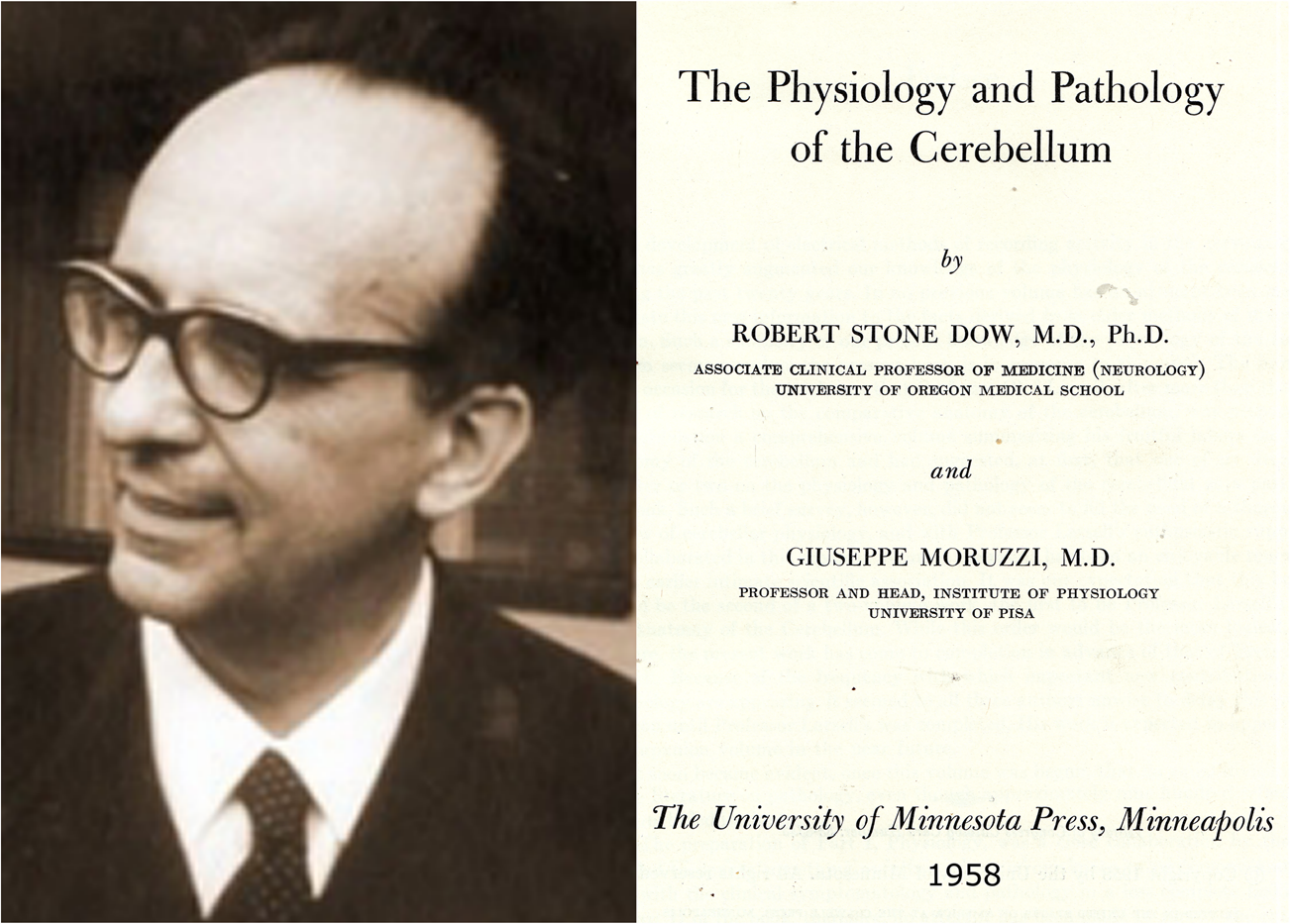
Giuseppe Moruzzi (1910–1986) and the front page of his book: Dow and Moruzzi [69]. Photograph reproduced from Levi-Moltalcini et al. [70]

**Fig. 23:**
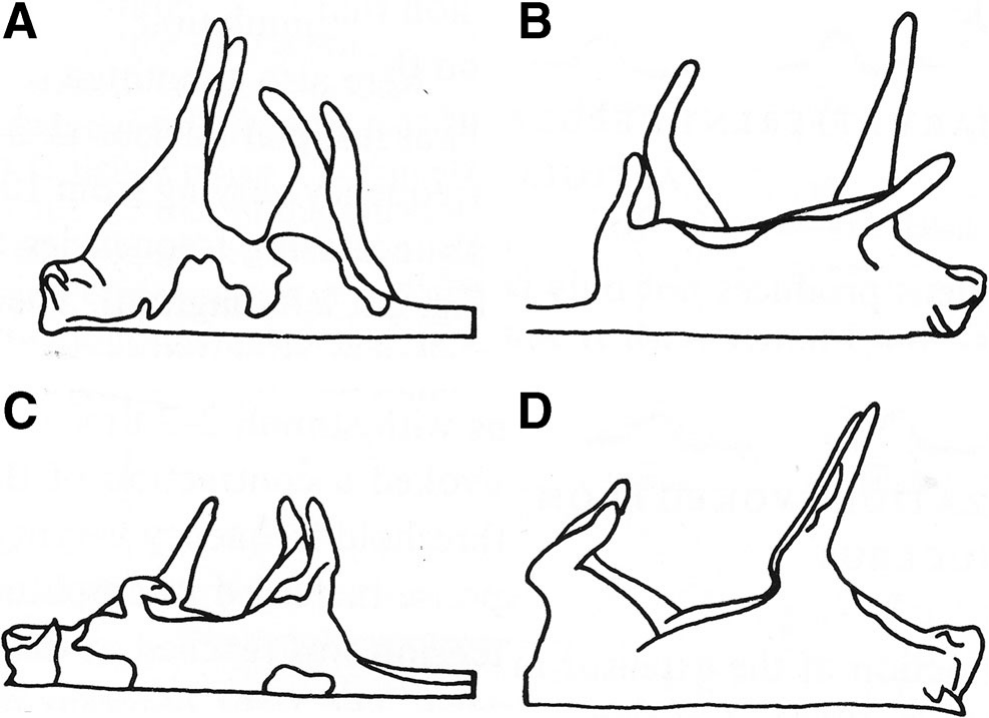
Effects of fastigial lesions on decerebrate rigidity. **a** Symmetrical distribution of extensor rigidity after precollicular decerebration. **b** Ipsilateral fastigial atonia after a lesion of the left rostral fastigial nucleus. **c** Contralateral fastigial atonia after a lesion of the right caudal fastigial nucleus. **d** Reappearance of symmetrical extensor rigidity after bilateral lesions of the caudal fastigial nucleus. Reproduced from Pompeiano [71]

As pointed out before, the fastigial nucleus projects bilaterally to ventral Deiters’ nucleus and the reticular formation through the uncinate and direct fastigiobulbar tracts (Fig. 11). The uncinate tract decussates in the cerebellar commissure and becomes applied to the contralateral rostral fastigial nucleus. Some of its fibers reach the intermediate gray and the anterior horn cells of the cervical cord [72, 73]. The Purkinje cell axons of the B zone on their way to Deiters’ nucleus are located immediately lateral to the fastigial nucleus. Lesions of the rostral fastigial nucleus, therefore, interrupt both uncinate tracts, and may include a proportion of the long corticofugal fibers of the B zone. Such a lesion also damages the direct fastigiobulbar tract. The uncinate tract is an excitatory system [74]. An excitatory effect of the direct fastigiobulbar tract on vestibular neurons was found by (Shimazu and Smith [75]). However, more recently, Bagnall et al. [76] produced convincing evidence in mice that this pathway is glycinergic and inhibits its target neurons. Moreover, it is not known whether the fastigial tracts terminate on excitatory or inhibitory neurons of the vestibular nuclei. Without further studies on the functional anatomy of the fastigial nucleus, these anatomical data are insufficient to explain Moruzzi’s observations on fastigial atonia.

The inhibitory nature of the Purkinje cells was discovered by Ito et al ([77], Fig. 24). According to Ito and Yoshida [78], they are responsible for the pronounced inhibitory effects of cerebellar muscle tone, as first demonstrated by Löwenthal and Horsley in 1897. A precise localization of these Purkinje cells in the anterior vermis was not yet possible [79], but they clearly occupy the lateral vermal B zone.

**Fig. 24:**
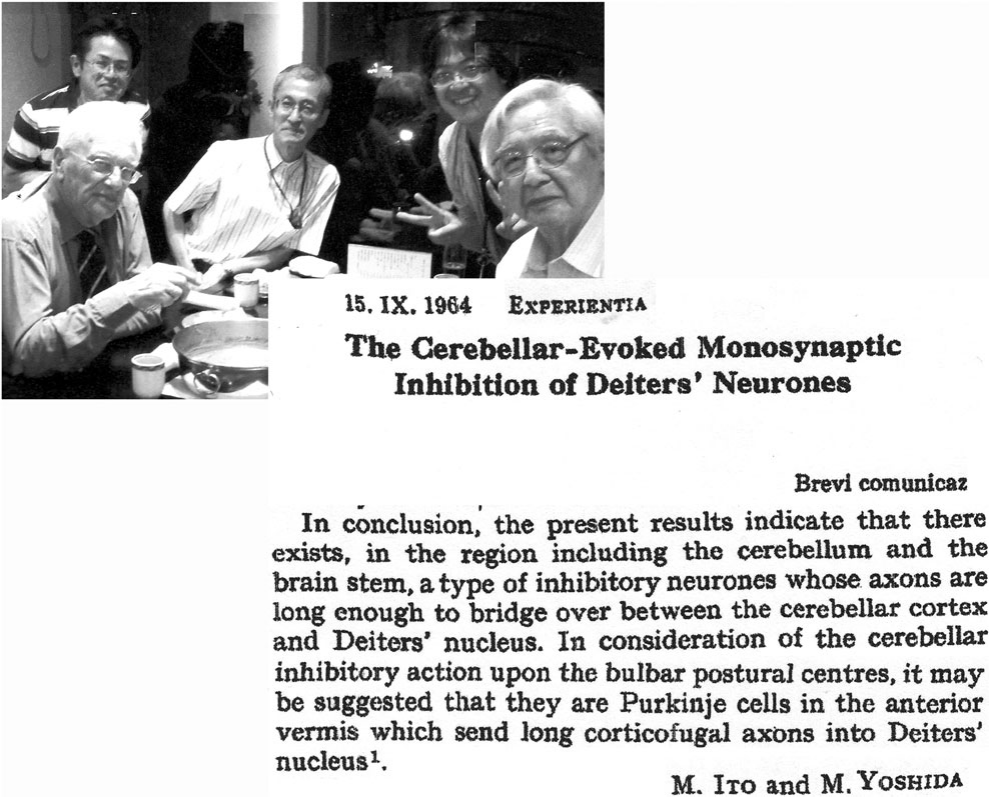
Conclusions of the paper of Ito and Yoshida [77] on the inhibitory nature of the Purkinje cells projecting to Deiters’ nucleus. The photograph shows Masao Ito as the host of an academic tour, presented to the author, at a dinner party in Tokyo in 2011, courtesy of Takeru Honda

Ito’s discovery of long-axon inhibitory neurons is the happy end of the story of cerebellar inhibition of decerebrate rigidity. Or is it? With stimulation of the anterior vermis, the inhibition by Purkinje cells of the B zone clearly dominates over effects of the vermal A and X zones on the fastigial nucleus. The fastigial nucleus certainly influences postural tone, by way of the vestibular nuclei and the reticular formation. Stimulation of the uncinate tract and the fastigial nucleus even was shown to evoke controlled locomotion, similar to stimulating the mesencephalic locomotion center. These effects are relayed by the reticulospinal tracts [80]. Detailed studies on the role of the fastigial nucleus in postural regulation are still lacking. Even less is known about the function of the X zone that projects through the interstitial cell groups to the spinal cord and the thalamus [81]. Deiters’ nucleus and its cerebellar and spinal connections have been a source for innovative studies of cerebellar function by many students. Knowing their involvement in postural systems and locomotion, the inhibitory nature of the Purkinje cells, the zonal organization of the climbing fibers, the somatotopical organization of the vermis, and the discovery of the microzones came about by using this cerebellar module as a model. As a fairly simple and one of the best-known modules, it may still challenge researchers, especially when studies would be combined with those of the other modules that constitute the anterior vermis.
